# Serotonin and Tryptophan Serum Concentrations in Shelter Dogs Showing Different Behavioural Responses to a Potentially Stressful Procedure

**DOI:** 10.3390/vetsci8010001

**Published:** 2020-12-24

**Authors:** Giacomo Riggio, Chiara Mariti, Valeria Sergi, Silvana Diverio, Angelo Gazzano

**Affiliations:** 1 Department of Veterinary Sciences, University of Pisa, 56124 Pisa, Italy; giacomo.riggio@phd.unipi.it (G.R.); sergi.valeria89@hotmail.it (V.S.); angelo.gazzano@unipi.it (A.G.); 2 Laboratory of Ethology and Animal Welfare (LEBA), Department of Veterinary Medicine, University of Perugia, via San Costanzo 4, 06126 Perugia, Italy; silvana.diverio@unipg.it

**Keywords:** behaviour, dog, serotonin, serum, tryptophan, stress, 5-HT, shelter

## Abstract

In mammals, serotonin (5-HT) levels depend on the availability of tryptophan (TRP). Low 5-HT concentrations have been linked to behavioural disorders in dogs. This study aimed at investigating possible differences in dogs’ serum TRP and 5-HT concentrations according to their behavioural response to a potentially stressful procedure. Thirty-nine physically healthy shelter dogs, 15 females and 24 males, mean age = 5.6 years, were categorized by a certified veterinary behaviourist according to their behavioural response to medical examination and blood collection, in: relaxation, stress signals, tension without growling, tension with growling, escape attempts, and aggression attempts. Extraction and quantification of 5-HT and TRP were performed using a HLPC method. Data were statistically analysed, applying Chi-square and Spearman tests. Results showed no significant difference in TRP (χ^2^ = 2.084, *p* = 0.555) nor 5-HT (χ^2^ = 0.972, *p* = 0.808) serum concentrations among different categories of dogs; however, some categories were underrepresented (relaxation = 20.5%, stress signals = 30.8%, tension without growling = 43.6%, tension with growling = 5.1%, escape attempts = 0%, aggression attempts = 0%). No correlation between serum TRP and 5-HT concentrations was found (ρ = 0.086, *p* = 0.602). Serum 5-HT levels do not seem to be associated with dogs’ behavioural response to a stressful situation nor with serum TRP concentrations. The relationship between serum TRP and 5-HT concentrations and behaviour needs further research.

## 1. Introduction

Serotonin (5-HT) is a monoamine neurotransmitter implicated in the regulation of a variety of physiological processes, cognitive functions, emotional states and behaviours in mammals [1,2,3]. For instance, low concentrations of 5-HT and its main metabolite, 5-hydroxyindoleacetic acid (5-HIAA), have been linked to aggressive and impulsive behaviour in rodents, humans and non-human primates [4,5,6,7]. On the contrary, in pigs and humans, high blood levels of 5-HT and 5-HIAA, respectively, have been associated with emotional aversive states, such as fear and anxiety [8,9].

Similar findings have been reported in dogs. In this species, low levels of serum 5-HT and CSF 5-HIAA have been linked to increased aggressive behaviour [10,11,12,13] and impulsivity [14], whereas high 5-HT plasma concentrations have been linked to anxious states [15].

Peripheral and central concentrations of 5-HT are strongly affected by the bioavailability of tryptophan (TRP). TRP is an essential amino acid that can be found in most protein-based foods and dietary proteins [16]. Alongside exerting a fundamental role in the biosynthesis of proteins, TRP is the obligatory substrate for the production of 5-HT in the gut and in the brain [17]. In the latter, the synthesis of 5-HT consists of a two-step process during which TRP is initially hydroxylated to 5-hydroxytryptophan by the enzyme TRP-hydroxylase and subsequently decarboxylated to 5-HT by the enzyme aromatic amino acid decarboxylase [18]. However, in order to pass the blood–brain barrier, TRP has to compete with large neutral amino acids (LNAAs), such as leucine, isoleucine, valine, tyrosine and phenylalanine for the same carrier mechanism [19]. Therefore, diets that increase the TRP/LNAAs ratio may have the power to increase TRP concentration in the brain and consequently increase central 5-HT levels [20,21]. Ultimately, variation of 5-HT brain concentration may affect an individual’s behaviour and emotional state.

In fact, there is some evidence that dietary TRP may reduce aggressive behaviour in rats [22] and primates [23], reduce fear and increase exploration in silver foxes [24], decrease self-injurious behaviour in primates [25], and positively affect the behavioural response to stressful stimuli in pigs [26]. However, studies on the effects of TRP supplemented diets on dog behaviour have led to inconsistent results. While some studies found TRP supplemented diets to reduce dogs’ aggressive [27] and stress-related behaviours [28], others found no behavioural effect on stereotypies [29] nor in anxious [30] or normally behaving dogs [31].

Overall, while a correlation between central TRP and 5-HT concentrations has been established [32], the mechanisms underlying the relationship between central and peripheral concentrations of these two molecules are still unclear, and so is the relationship between TRP and 5-HT levels in circulating blood. Indeed, if a correlation between TRP and 5-HT peripheral concentrations was to be confirmed, less invasive, less expensive, and less time-consuming procedures could be implemented to measure the effects of this amino acid on an individual’s hormonal status. Unfortunately, those few studies on dogs that simultaneously assessed TRP and 5-HT concentrations have led to different results. In a pilot study on phobic dogs fed with a carbohydrate dissociated diet—a diet in which one of the daily meals is composed of only carbohydrates with the aim of stimulating insulin secretion and consequently increasing the muscle uptake of LNAAs other than the albumin–bound fraction of TRP—Gazzano et al. [33] found no correlation between TRP and 5-HT serum concentrations over time. On the contrary, DeNapoli [27] found a positive correlation between these two molecules in aggressive dogs’ blood.

Considering the conflicting findings on the relationship between TRP and 5-HT peripheral levels, as well as the evidence on the effects of 5-HT on dog behaviour, the aim of this study was twofold: (1) to investigate the possible correlation between TRP and 5-HT serum concentrations in dogs that, differently from similar previous studies, were on a normal feeding regimen; (2) to assess potential differences in dogs’ TRP and 5-HT peripheral concentrations in relation to their behavioural response to a potentially stressful procedure, which in this case was represented by a veterinary examination [34,35].

## 2. Materials and Methods

### 2.1. Subjects

Thirty-nine mix-breed dogs, 15 females (11 spayed) and 24 males (all intact), their age ranging from 7 months to 14 years (mean age = 5.6 years), participated in the study. Recruited subjects had been living in a shelter for a minimum of 1 month to a maximum of 9 years (median = 12 months). All dogs were recruited from two different facilities: 10 of them were housed at the Pisa public shelter, while 29 were housed at the Lucca public shelter. General management and routine procedures did not substantially differ between facilities, since they were both run by the same non-profit organization. All dogs were housed in either single or double kennels, with both indoor and outdoor spaces available and were fed twice a day, at 8 a.m. and 4 p.m., with commercial dry food. None of the dogs underwent a comprehensive behavioural evaluation prior to the experiment; however, those dogs that displayed aggressive behaviour towards humans—that could jeopardize the safety of the people involved in the study and/or that would require sedation in order to be examined—as well as those presenting signs of physical disorders (i.e., neurological, orthopaedical, dermatological) or injuries that could affect their behavioural response to manipulation, were excluded from the study. All dogs had undergone at least one prior medical evaluation, since they would be examined at the time of their arrival and, in the absence of diseases, before annual vaccination.

### 2.2. Experimental Setting and Procedure

The study was carried out over a 5-week period between November and December 2019. On the day of the procedure, the selected dog was taken out of the kennel by a shelter volunteer and brought to the examination room within the facility, where a veterinarian immediately performed a general medical exam to ensure the dog was in good health. The same veterinarian performed all examinations in both facilities. The whole procedure was part of the periodical medical check-up to assess the dogs for parasitic diseases and general health status. It lasted approximately 10 min for every dog and consisted of the following steps: putting a muzzle on the dog, placing the dog on the examination table, evaluation of conjunctival mucosa, palpation of lymph nodes, evaluation of ear canals, measurement of rectal temperature, auscultation of heart and lungs. After the examination, a venous blood sample was collected. A certified behaviourist, which was the same for all dogs, observed and scored their behavioural response to containment, manipulation and blood collection, starting the observation when the muzzle was put on until the moment it was taken off, right after the blood sampling. Dogs were scored based only on the behaviours displayed during this time interval, since the actual examination has been reported to be the most frightening phase of the veterinary visit [36], and therefore the most likely to provoke a response. Each dog could receive one of the following scores: 1 = relaxation, 2 = stress signals (lip-licking, head-turning, crouched posture, trembling), 3 = tension without growling, 4 = tension with growling, 5 = escape attempts, and 6 = aggression attempts. In case dogs displayed behaviours belonging to two different categories (i.e., stress signals and aggression attempts), the veterinary behaviourist classified them based on their highest score. The scoring system was based on and modified from Mills et al. [37].

### 2.3. Blood Collection, Storage and Analysis

Blood was collected from the cephalic vein after 3–4 h from the dog’s morning meal in order to determine the levels of 5-HT and total TRP. Blood samples (4 mL) were left to coagulate at room temperature for 30/60 min, then centrifuged in ALC 4237R Refrigerated Centrifuge (ALC International S.r.l., Milan, Italy) at 7000 rpm for 20 °C to 4 °C to obtain the serum. The serum was divided into aliquots from 200 µL and frozen until the time of analysis, which was performed 6 months after the sampling, at the latest.

The extraction and quantification of 5-HT and TRP in serum samples were performed following an HPLC method, based on fluorimetric detection, with the same methods described in Gazzano et al. [33]. This method was based on Bearcroft et al. [38] and Atkinson et al. [39] and slightly modified as follows: 200 µL HCLO₄ 4% *v*/*v* containing 2 mM EDTA was added to 200 µL of serum or standard solution to precipitated proteins; the extract was mixed and centrifuged at 13,000 rpm in micro centrifuge (microCENTRIFUGETTE^®^ 4214, ALC International S.r.l., Milan, Italy) for 3 min. Then, 50 µL of Supernatant was taken with MICROLITER™ Syringes #705 and 20 µL injected into HPLC for analysis.

HPLC analyses were performed using an RP Gemini C18 column (250 mm × 4.6 mm, 5 μm) (Phenomenex, Torrance, CA, USA) and a Jasco HPLC apparatus (Jasco Corporation, Ishikawa-Machi Hachioji-Shi, Tokyo, Japan) equipped with 2 gradient pumps (PU-1580), a mixer unit (HG-2080-03) and a fluorescence detector (FP-920).

The mobile phase consisted of methanol (CH₃OH) and ammonium acetate (CH₃COONH₄) 100 mM (20:250 *v*/*v*), pH 4.5, degassed and filtered with 0.2 µm diameter filters and eluted at a flow rate of 0.800 mL/min.

The fluorescence detector was set at 290 nm excitation wavelength and 337 nm emission wavelength. Data were acquired using Jasco Borwin 1.5.0 software (Jasco Corporation, Ishikawa-machi Hachioji-shi, Tokyo, Japan). The interface between chromatography instruments and a PC based data acquisition is the JMBS electronic interface box HERCULE 2000 VI.0.

Serotonin Creatinine sulfate monohydrate and L-tryptophan (TRP) were purchased from Sigma-Aldrich Inc. (Saint Louis, MO, USA).

Stock solution (10 mM) of 5-HT and stock solution (100 mM) of TRP were prepared in 10 mL HClO₄ 10%, divided in aliquots of 1 mL and stored at −20 °C. Diluted standard solutions in HClO₄ 4% were prepared daily and employed to identify chromatographic peaks and to calculate calibration curves.

### 2.4. Statistical Analysis

Data were statistically analysed by using SPSS^®^ STATISTICS 17.0. Spearman Rho test was performed to analyse the possible correlation between TRP and 5-HT serum concentrations in the whole sample of dogs. Chi-square test was applied to analyse possible differences in both TRP and 5-HT concentrations among groups of dogs with different behavioural scores in response to the experimental procedure.

## 3. Results

Based on the behavioural classification, 9 dogs (23.1%) remained relaxed during the procedure, 12 (30.8%) displayed stress signals, 16 (41.0%) appeared tense, but did not growl, and 2 (5.1%) appeared tense and growled. None of the subjects attempted to escape or manifested overt aggression towards the veterinarian performing the examination and the blood collection.

Serotonin serum concentrations ranged from 29.928 to 430.186 ng/mL, with a median value equal to 183.3845 ng/mL. Tryptophan concentrations ranged from 58.335 to 157.304 µg/dL, with a median value of 106.836 µg/dL.

**Figure 1:**
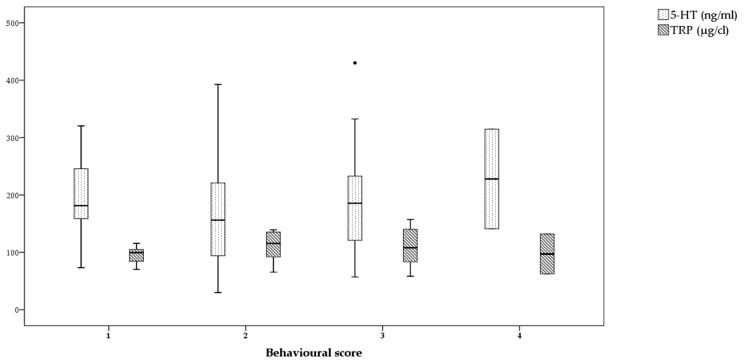
Tryptophan (TRP) (striped boxes) and serotonin (5-HT) (dotted boxes) concentrations (TRP: µg/cL, 5-HT: ng/mL) in dogs showing the following behavioural scores: relaxation (1), displaying stress signals (2), showing tension without growling, (3) and showing tension with growling (4) during the medical examination.

Statistical analysis revealed no correlation between TRP and 5-HT serum concentrations (ϱ = 0.086, *p* = 0.602). Figure 1 shows the different serum concentrations of both TRP and 5-HT found in dogs with different scores. In no category of dogs can a common trend between the analytes be observed. Furthermore, no significant differences in either TRP (χ^2^ = 2.084, *p* = 0.555) nor 5-HT (χ^2^ = 0.972, *p* = 0.808) serum concentrations were found between dogs that remained relaxed (TRP: median = 99.52800, min–max = 70.385–115.660; 5-HT: median = 181.37201, min–max = 73.103–320.319), dogs that displayed signs of stress (TRP: median = 115.56432, min–max = 65.483–139.347; 5-HT: median = 156.12664, min–max = 29.928–392.737), dogs that appeared tense but did not growl (TRP: median = 108.06257, min–max = 58.335–157.304; 5-HT: median = 185.62264, min–max = 56.950–430.186), and those that were tense and did growl (TRP: median = 97.22486, min–max = 62.545–131.905; 5-HT: median = 227.85393, min–max = 14.994–314.714).

## 4. Discussion

In the present study, no significant correlations were found between TRP and 5-HT serum concentrations in our sample of shelter dogs. This result is in accordance with findings from a previous pilot study by Gazzano et al. [33]. Considering that positive correlation reported between TRP and 5-HT in the brain has been demonstrated [32], a possible explanation for this lack of correlation at peripheral level may lie in the role that the blood-brain barrier plays in regulating the passage of TRP from peripheral to central circulation and that of 5-HT in the opposite direction.

As for TRP, approximately 70–90% of this amino acid in blood circulation is bound to albumin [17,19]. In order to cross the hematoencephalic barrier, TRP must not be bound to albumin [17,40]. Therefore, while circulating free TRP may directly bind to the blood–brain barrier for its transportation to the brain, the albumin-bound fraction must first be released from the protein binding sites. This latter process occurs because of TRPs greater affinity to the binding sites of the hematoencephalic barrier than those of albumin [41]. As equilibrium mechanisms are rapidly activated, an increase in free TRP and brain intake may reflect in a reduction in total TRP (free + albumin-bound) [41]. Different values of their absolute concentrations and their relative percentages may have a different physiological significance [41,42]. Furthermore, TRP competes with other LNAAs for its transportation across the barrier by means of a carrier protein [19]. This is likely the reason why a non-specific increase in protein intake does not produce consistent effects on dog behaviour [20,27,43], whereas a diet aimed to increase the TRP/LNAAs ratio, by either TRP supplementation or through dissociated carbohydrate-based diets [19], seems to be effective at increasing 5-HT central concentrations [21], and ultimately at modifying behaviour. Therefore, the link between peripheral TRP and 5-HT may be better understood by measuring both free and total TRP serum concentrations and TRP/LNAAs ratio, rather than just one of the formers [41,42], as was performed in this and other studies.

As for the relationship between 5-HT central and peripheral levels, studies performed on humans and laboratory animals led to conflicting findings. For instance, Pietraszek et al. [32] found no correlation between brain and blood 5-HT levels in laboratory mice, whereas other studies reported good correlations between 5-HT levels in CSF and peripheral matrices, such as plasma [44,45], whole blood [46], platelets [44], in rats, humans and non-human primates. For a long time, it was accepted that central 5-HT could not cross the blood-brain barrier [47]. Therefore, peripheral 5-HT was believed to origin from synthesis in peripheral sources, mainly the enterochromaffin cells in the gut [47]. On the contrary, more recent studies have been able to identify serotonin transporters on the endothelial cells of blood vessels [48,49,50] in the hematoencephalic barrier and demonstrate that 5-HT can actually translocate from the brain into the blood flow [47]. Although these findings suggest that peripheral 5-HT may partially have central origin, there may not be a linear and direct correlation between fluctuations of central and peripheral 5-HT levels.

In the present study, we observed no significant differences in TRP serum concentrations between dogs displaying different behavioural responses to a potentially stressful procedure, which in this specific case was represented by medical examination and blood sampling. Low serum TRP concentrations have been linked to aggressive behaviour in several mammal species, including dogs [23,27,51,52]. Previous studies, however, majorly rely on the supplementation or depletion of TRP through diet manipulation. In our case, no special diet was given to the dogs; hence, variation in TRP levels may have not been substantial enough to affect behaviour. Overall, it is generally accepted that TRP’s effect on behaviour is strictly dependent on its capability to cross the blood–brain barrier and increase 5-HT brain levels [17]. According with the results obtained by Rayment et al. [53] in a recent investigation on the correlation between 5-HT and behaviour, and consistently with our findings on TRP, in this study we did not observe any significant difference in the serum levels of this neurotransmitter among dogs showing distinct behavioural responses.

On the contrary, some previous studies found different serum 5-HT concentrations between normally behaving dogs and dogs displaying fearful and aggressive behaviours [10,11,12,15]. As Rayment et al. [53] suggest, methodological factors may explain different results. Firstly, all of these studies involved dogs with aggressive or anxiety behaviour problems that were severe enough to require behavioural consultation. This is particularly important as previous research suggests that 5-HT levels may significantly affect behaviour only in those subjects that are susceptible to mood disorders [54]. Instead, in our study, none of the dogs displayed intense aggressive or fearful behaviours (even in a potentially stressful situation), nor did they show any response that could suggest an underlying behavioural disorder. The lack of overt display of aggressive behaviour may be due to our decision to exclude dogs with known history of aggressive behaviour towards people for both safety and procedural reasons. Furthermore, it must be pointed out that none of the dogs involved in the present study underwent a comprehensive behavioural evaluation, which may have helped draw a clearer picture on their general behavioural characteristics beyond the specific context of the experimental procedures. Having a more heterogeneous sample of dogs in terms of behaviour may be necessary in order to reveal possible differences in 5-HT concentrations.

Furthermore, 5-HT concentrations may vary substantially in relation to the matrix used for its quantification. In fact, plasma 5-HT levels have been found to be up to 40 times lower than those reported for serum in dogs [55]. Serum serotonin median concentration found in this study (183.4 ng/mL) falls within the reference range of median 5-HT serum values previously observed in clinically healthy dogs with no reported history of behavioural disorders (32.5–509.8 ng/mL) [10,11,12,56,57]. However, this range is very broad and unlikely to be useful in clinical applied settings [58]. As suggested by Alberghina et al. [56], the width of this range may be explained by the lack of standardization of preanalytical factors across studies, such as feeding regimen of the experimental subjects [59], time of the sampling [59,60,61], storage duration of the sample and temperature fluctuations during storage [58], which have been reported to affect serotonin concentrations in mammal species.

This study has limitations that need to be addressed. Firstly, our sample of dogs was not heterogeneous enough to represent all possible behavioural categories. Specifically, we did not observe any severe fearful or aggressive behaviour. This, of course, may have affected our findings, although it may more closely mirror the situation in most of the canine population. Secondly, although veterinary medical examination has been demonstrated to produce an acute stress response in dogs [62,63], in this study we did not assess physiological parameters of stress, such as cortisol, for instance. This may have provided us with additional and relevant information on the hormonal picture induced by the experimental procedure, especially considering the effects of corticosteroids on TRP metabolism, and consequently on 5-HT concentrations [33,53,64].

## 5. Conclusions

In the present study, no correlation between serum TRP and 5-HT levels were found in our sample of shelter dogs. Since it is widely accepted that TRP and 5-HT concentrations are correlated in the brain, our negative finding may be at least partially explained by the role of the blood-brain barrier, regulating the passage of TRP from peripheral to central circulation and that of 5-HT in the opposite direction. Considering the conflicting literature on the topic, further research is needed to clarify the kinetics of both molecules across the hematoencephalic barrier, as well as the relationship between each molecule’s central and peripheral levels. Furthermore, in this study no differences were found in dogs’ serum TRP and 5-HT concentrations in relation to their behavioural response to medical examination and blood collection. Considering the differences in some methodological aspects (e.g., behavioural features of the experimental subjects, behavioural assessment methods, peripheral matrices for 5-HT quantification, 5-HT storage time and procedure) across previous studies on 5-HT, as well as earlier findings on the link between low serum 5-HT and behavioural disorders in dogs and other mammal species, our results should not suggest that such link is instead non-existent. However, they do suggest that greater consistency on the methodological approach across studies may be necessary in order to be able to draw clearer conclusions on the relationship between peripheral 5-HT levels in dogs and their behaviour.

## Data Availability

Data are available on request from the corresponding author.
